# Supplementary material for: Solar-Driven Thermocatalytic Synthesis of Octahydroquinazolinone Using Novel Polyvinylchloride (PVC)-Supported Aluminum Oxide (Al2O3) Catalysts
Source: Materials (Basel). 2023 Apr 2;16(7):2835. doi: 10.3390/ma16072835 (PMC10096424; doi:10.3390/ma16072835)
Supplement: Supplementary file 1 [file materials-16-02835-s001.zip › materials-2216971-supplementary.pdf]

Supporting Information

Solar-Driven Thermocatalytic Synthesis of Octahydroquinazolinone Using Novel Polyvinylchloride (PVC)-Supported Aluminum Oxide (Al_2O_3) Catalysts

Abdulrahman I. Alharthi [1], Mshari Alotaibi [1], Amani M. Alansi [2], Talal F. Qahtan [3], Imtiaz Ali [4], Matar. N. Al-Shalwi [5] and Md. Afroz Bakht [1,*]

[1] Chemistry Department, College of Science and Humanities Studies, Prince Sattam Bin Abdulaziz University, P.O. Box 83, Al-Kharj 11942, Saudi Arabia
[2] Chemistry Department, King Saud University, Riyadh 12372, Saudi Arabia
[3] Physics Department, College of Science and Humanity Studies, Prince Sattam Bin Abdulaziz University, P.O. Box 83, Al-Kharj 11942, Saudi Arabia
[4] Preparatory College, Prince Sattam Bin Abdulaziz University, P.O. Box 83, Al-Kharj 11942, Saudi Arabia
[5] Electrochemical Sciences Research Chair (ESRC), Department of Chemistry, College of Science, King Saud University, P.O. Box 2455, Riyadh 11451, Saudi Arabia
* Correspondence: m.bakht@psau.edu.sa; Tel.: +966-55375-3763

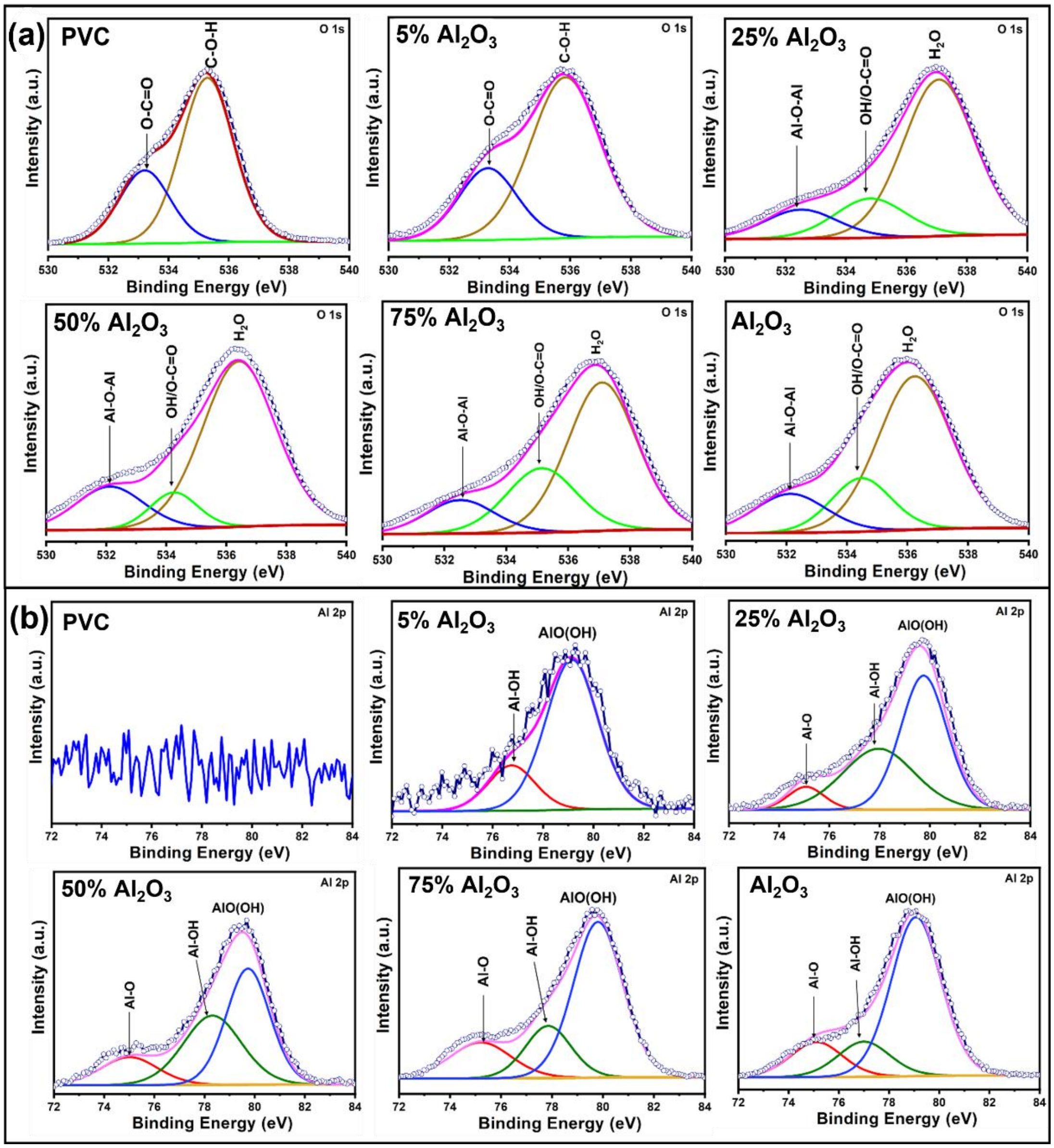

Figure S1. O1s XPS spectra (a) and C 1s (b), and Cl 2p spectra of Al_2O_3, PVC, and Al_2O_3/PVC samples containing different aluminum trioxide amounts as indicated.

Table S1 the ratios of the electronic state of the elements for the as-prepared Al_2O_3/PVC catalysts

Catalyst/Atomic ratio of elements	C	Cl	O	Al	Ca	S
**PVC**	61.85	8.73	29.42	0	0	0
**5% Al_2O_3**	56.15	10.71	28.78	4.01	0	0
**25% Al_2O_3**	34.07	7.27	40.89	15.09	2.04	0.01
**50% Al_2O_3**	20.45	4.04	54.92	18.44	2.12	0.03
**60% Al_2O_3**	14.89	2.64	56.53	22.38	2.95	0.61
**75% Al_2O_3**	12.77	2.13	53.90	28.25	2.13	0.04
**Al_2O_3**	7.04	0	63.46	27.38	2.08	0.03

NMR Spectra of Synthesized Compounds 4a-h

4a-HNMR

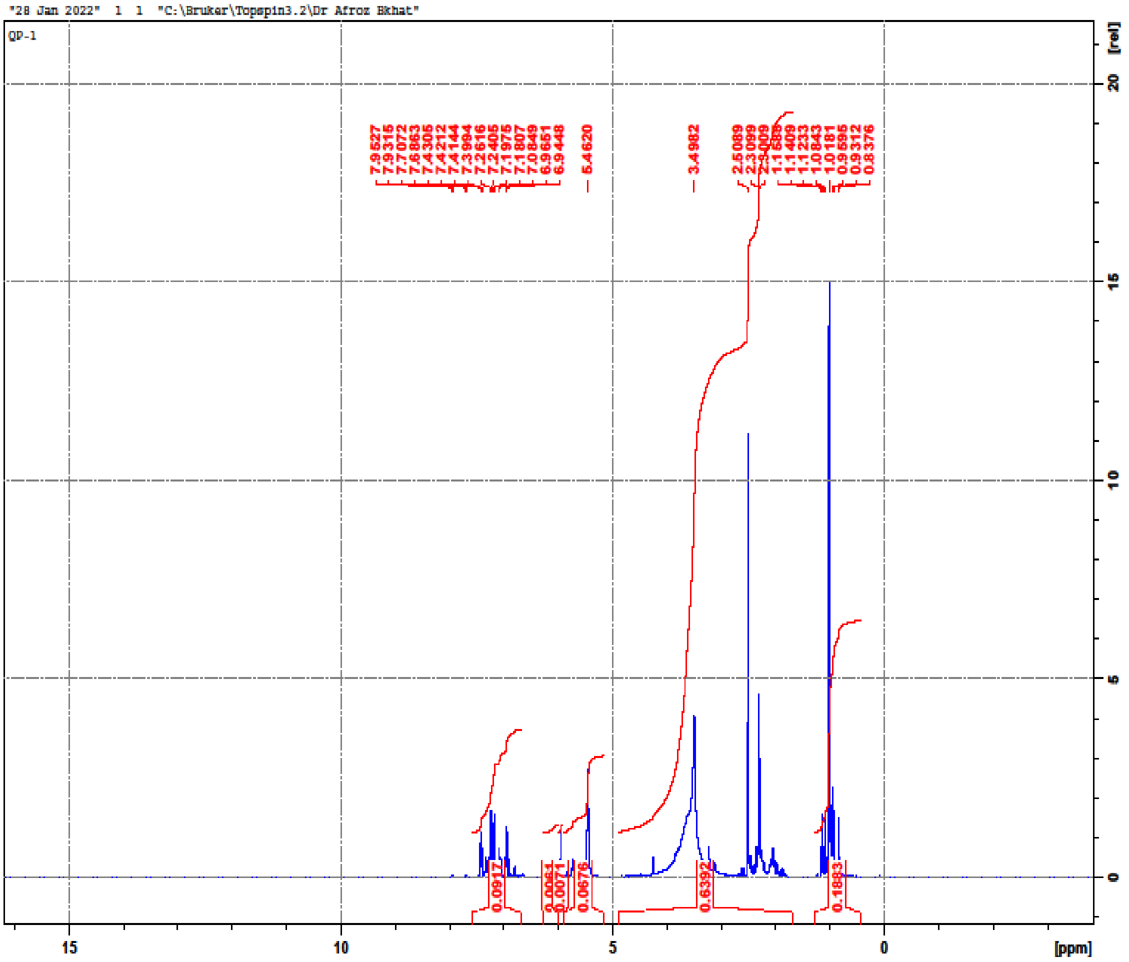

4a-^{13}CNMR

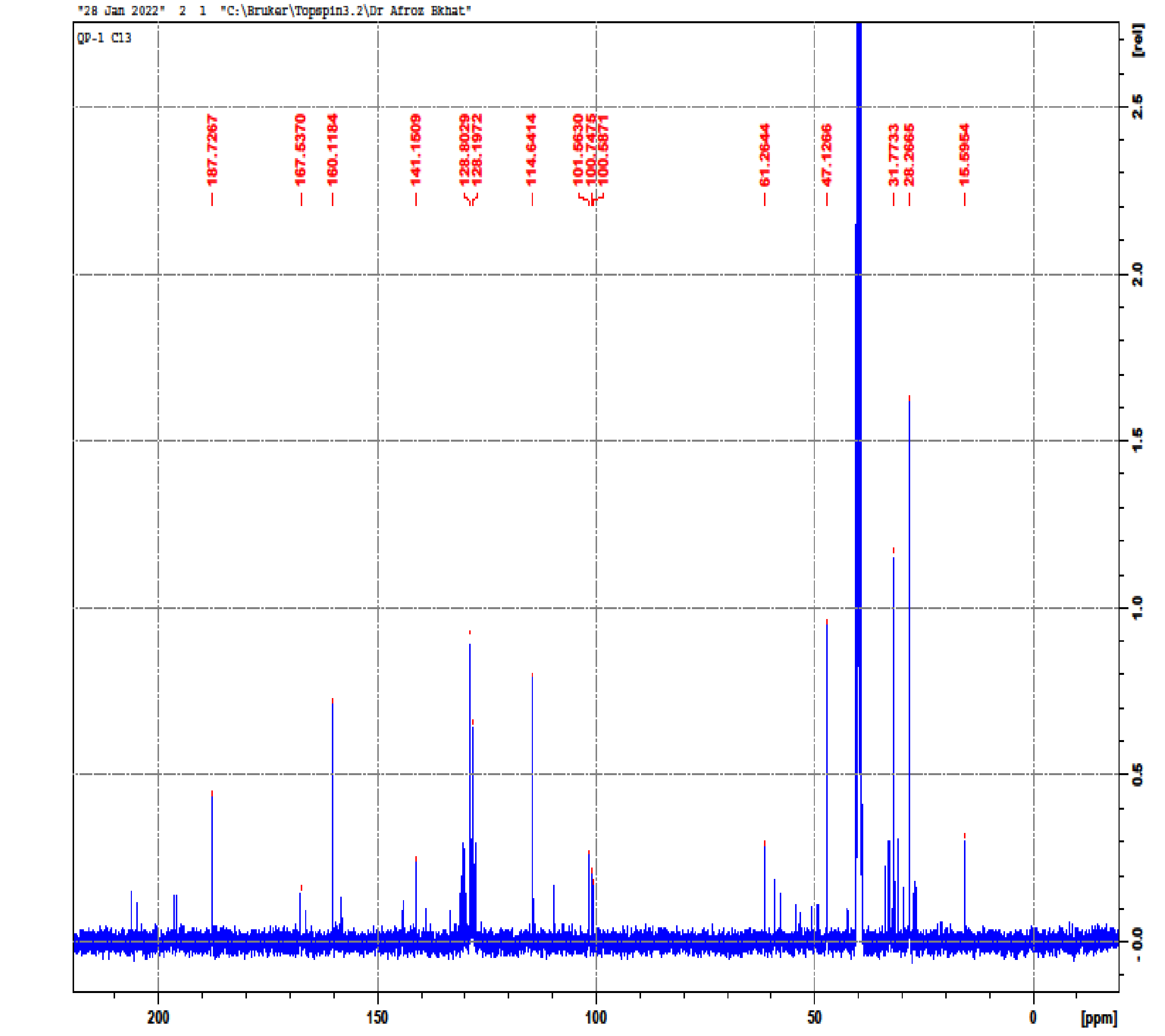
"28 Jan 2022" 2 1 "C:\Bruker\Topspin3.2\Dr Afroz Bkhat"
QP-1 C13
187.7267
167.5370
160.1184
141.1509
128.8029
128.1972
114.6414
101.5630
100.7475
100.5871
61.2644
47.1266
31.7733
28.2665
15.5954
[rel]
[ppm]

4b-^{13}CNMR

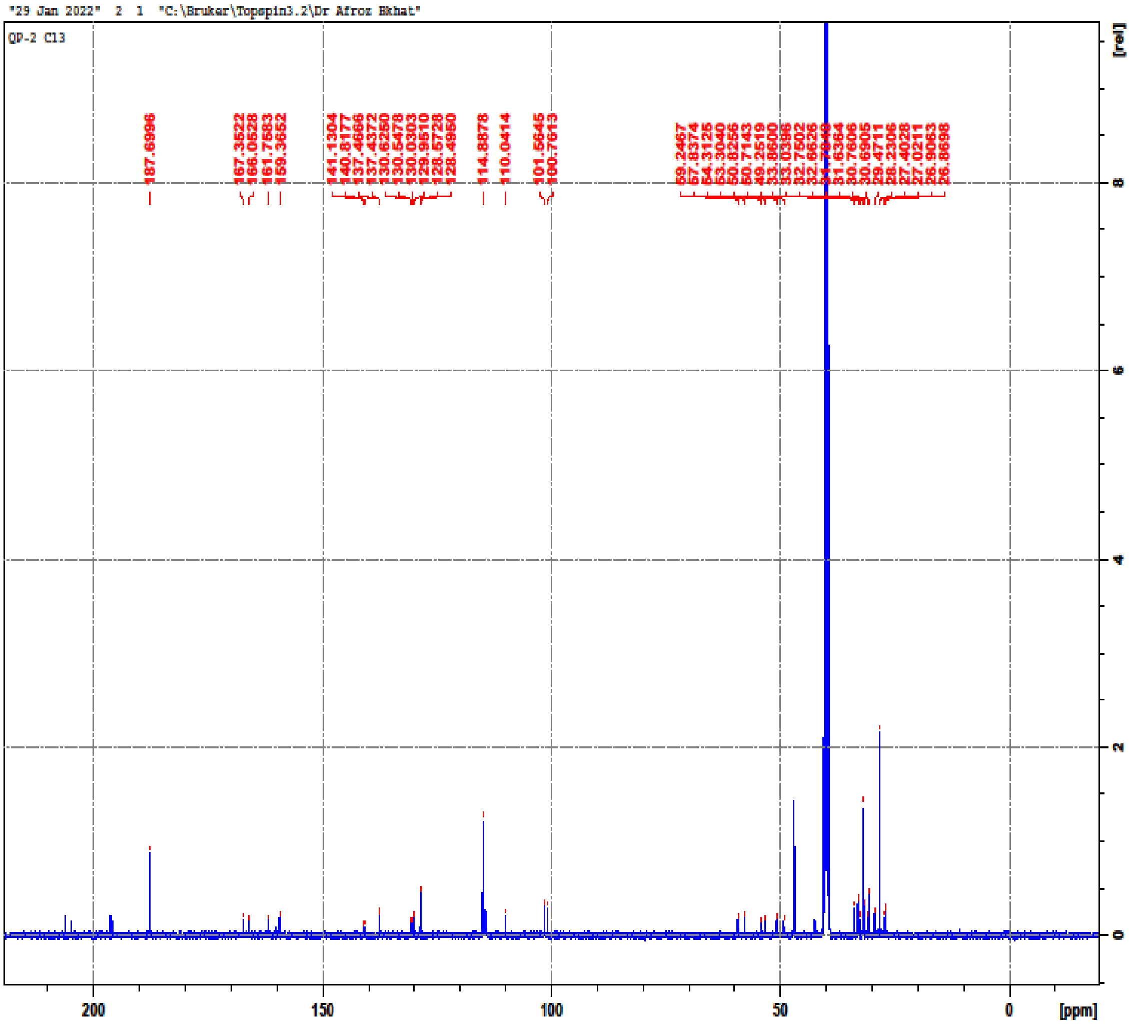
"29 Jan 2022" 2 1 "C:\Bruker\Topspin3.2\Dr Afroz Bkhat"
QP-2 C13
187.6996
167.3522
166.0528
161.7583
159.3652
141.1304
140.8177
137.4666
137.4372
130.6250
130.5478
130.0303
129.9510
128.5728
128.4950
114.8878
110.0414
101.5645
100.7613
59.2467
57.8374
54.3125
53.3040
50.8256
50.7143
49.2519
33.8600
33.0386
32.7502
32.6626
31.6364
30.7606
30.6905
29.4711
28.2306
27.4028
27.0211
26.9063
26.8698
[rel]
8
6
4
2
0
200
150
100
50
0
[ppm]

4b-HNMR

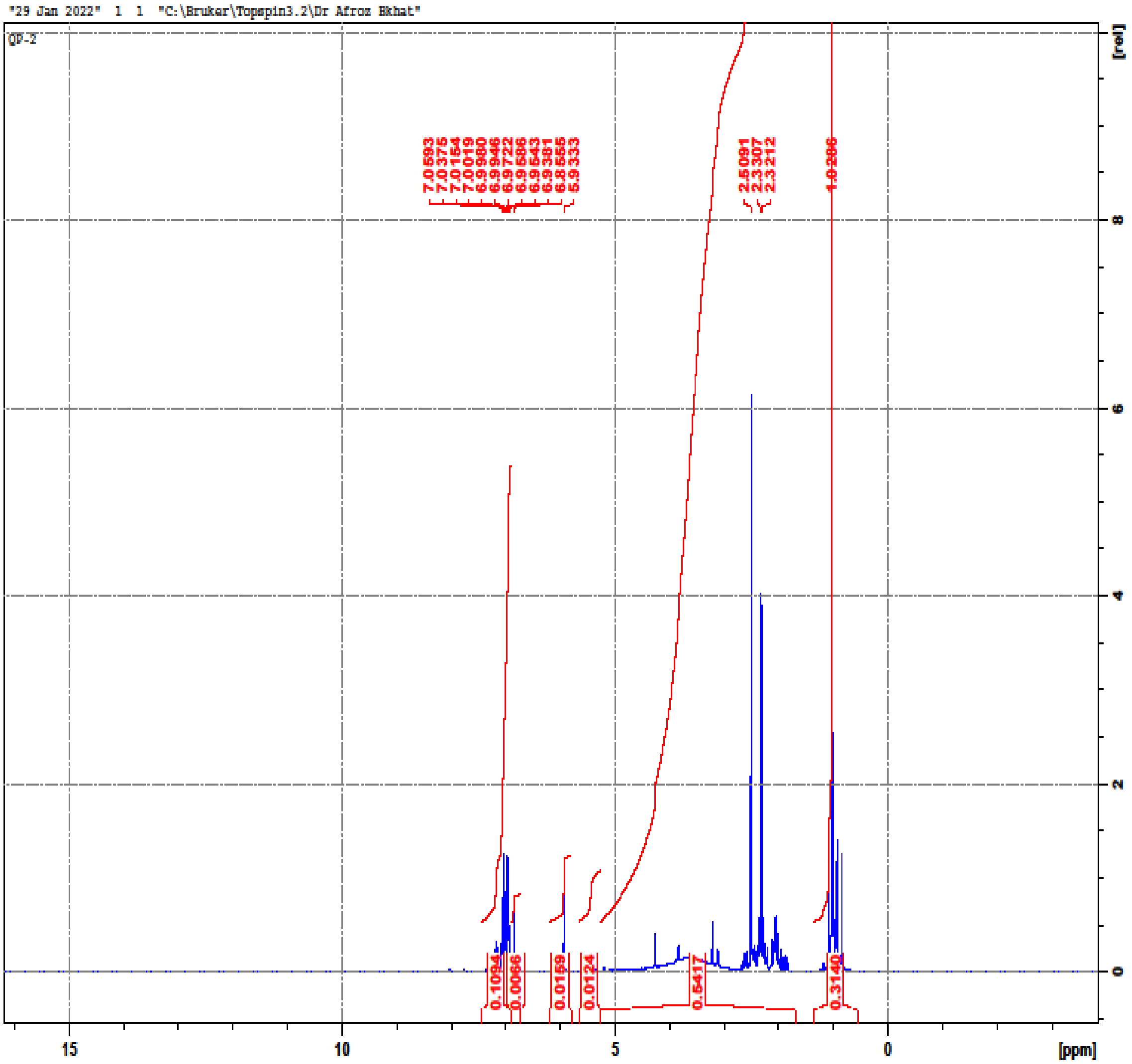
"29 Jan 2022" 1 1 "C:\Bruker\Topspin3.2\Dr Afroz Bkhat"
QP-2
7.0593
7.0375
7.0154
7.0019
6.9980
6.9946
6.9722
6.9586
6.9543
6.9381
6.8555
5.9333
2.5091
2.3307
2.3212
1.0286
0.1094
0.0066
0.0159
0.0124
0.5417
0.3140
15
10
5
0
[ppm]
[rel]
8
6
4
2
0

4c-^{13}CNMR

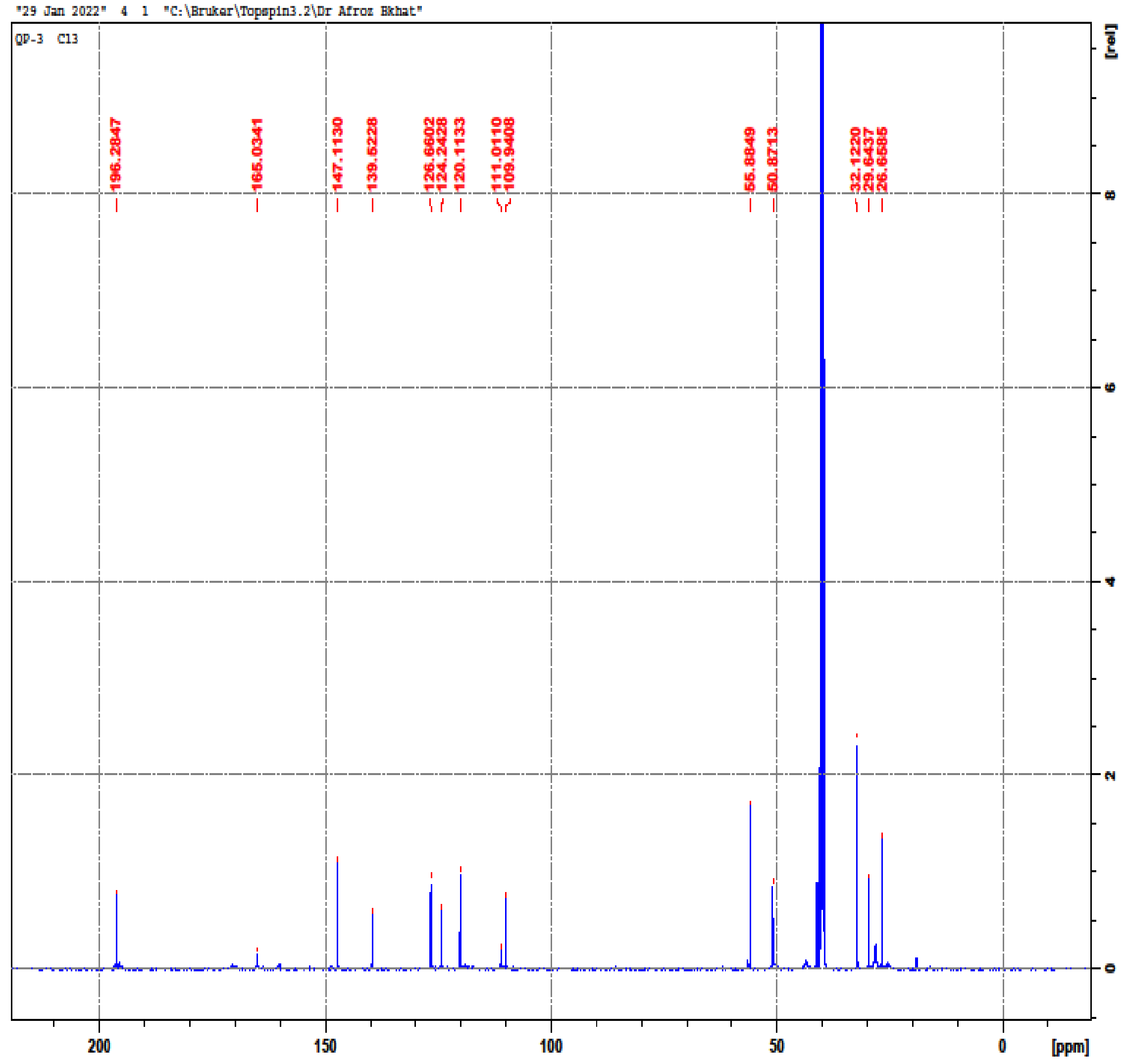
"29 Jan 2022" 4 1 "C:\Bruker\Topspin3.2\Dr Afroz Bkhat"
QP-3 C13
196.2847
165.0341
147.1130
139.5228
126.6602
124.2428
120.1133
111.0110
109.9408
55.8849
50.8713
32.1220
29.6437
26.6585
[rel]
200
150
100
50
0
[ppm]

4c-HNMR

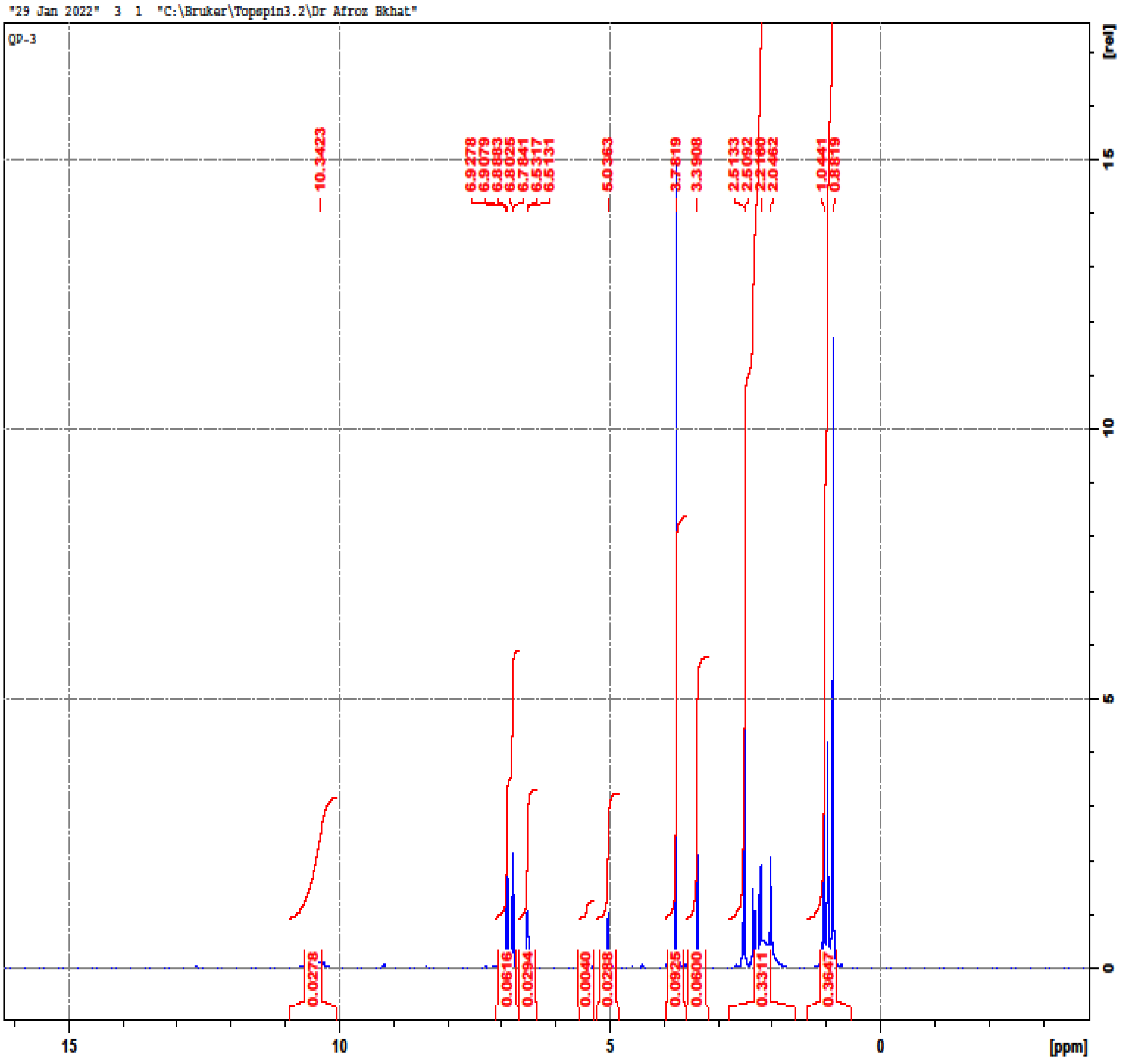
"29 Jan 2022" 3 1 "C:\Bruker\Topspin3.2\Dr Afroz Bkhat"
QP-3
10.3423
6.9278
6.9079
6.8883
6.8025
6.7841
6.5317
6.5131
5.0363
3.7619
3.3908
2.5133
2.5092
2.2160
2.0462
1.0441
0.8819
0.0278
0.0616
0.0294
0.0040
0.0288
0.0926
0.0600
0.3311
0.3647
[rel]
[ppm]

4d-^{13}CNMR

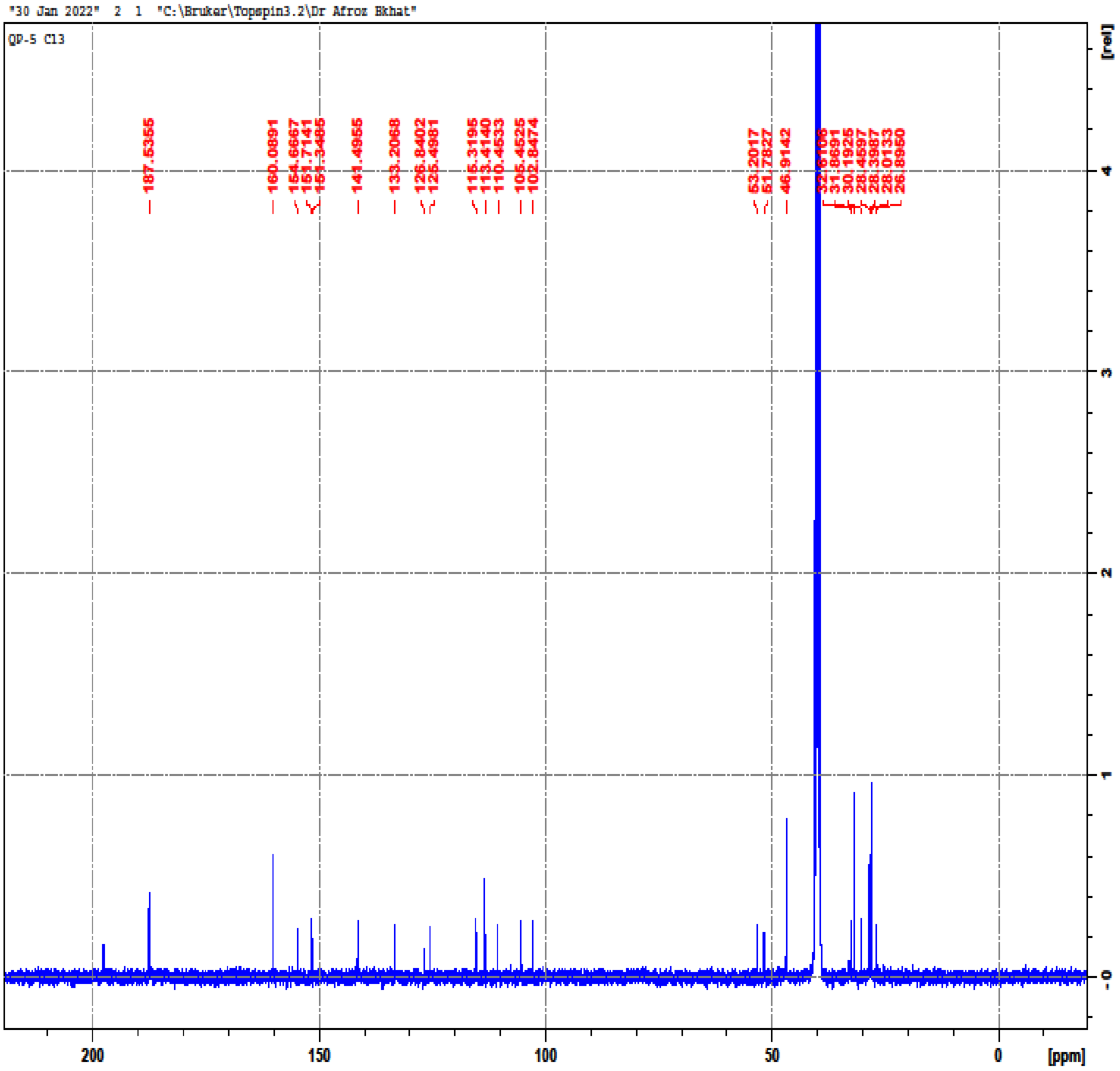
"30 Jan 2022" 2 1 "C:\Bruker\Topspin3.2\Dr Afroz Bkhat"
QP-5 C13
187.5355
160.0891
154.6667
151.7141
151.3485
141.4955
133.2068
126.8402
125.4981
115.3195
113.4140
110.4533
105.4525
102.8474
53.2017
51.7827
46.9142
32.6106
31.8691
30.1925
28.4597
28.3987
28.0133
26.8950
[rel]
200
150
100
50
0
[ppm]

4d-HNMR

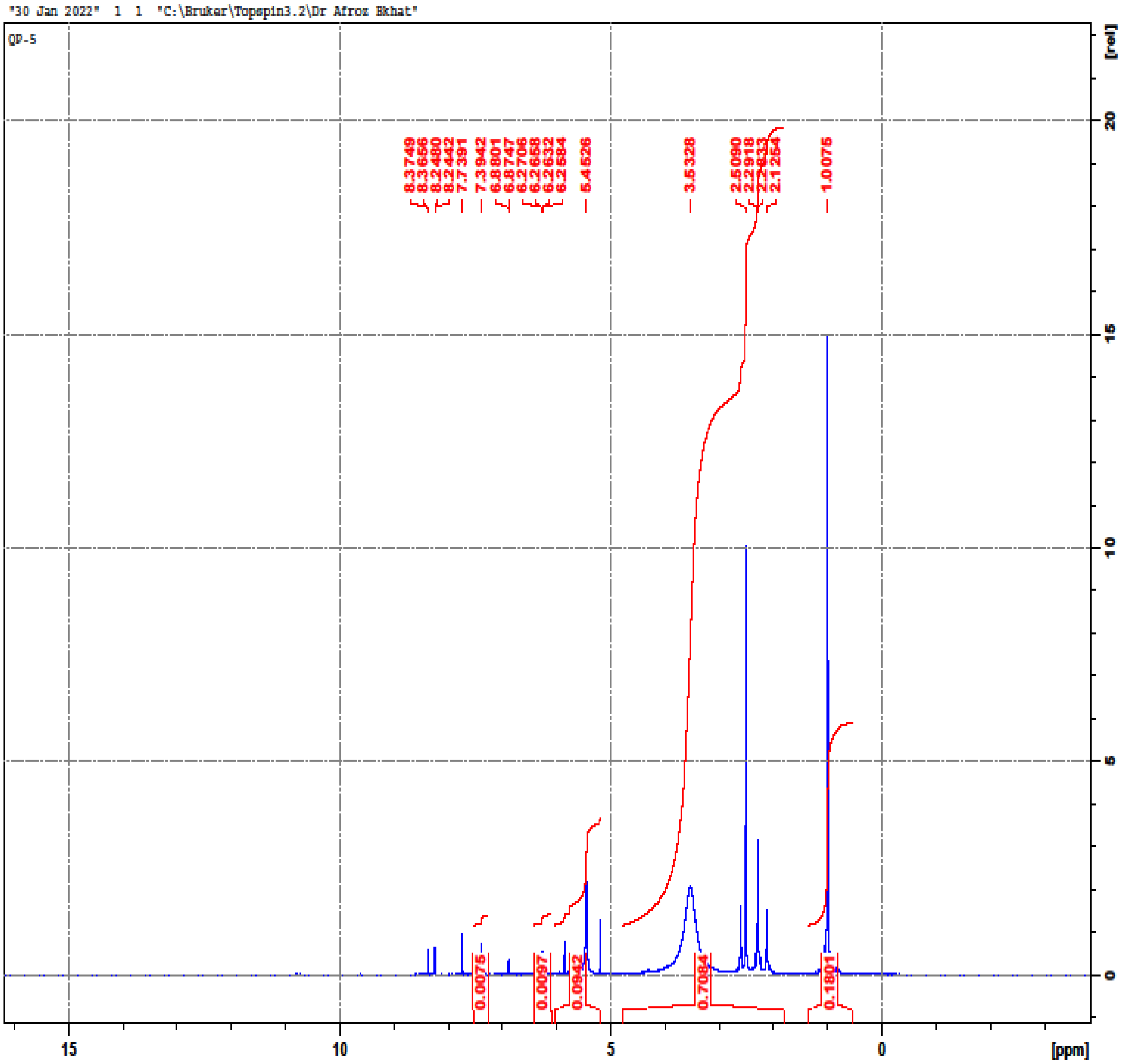
"30 Jan 2022" 1 1 "C:\Bruker\Topspin3.2\Dr Afroz Bkhat"
QP-5
8.3749
8.3666
8.2480
8.2442
7.7391
7.3942
6.8801
6.8747
6.2706
6.2658
6.2632
6.2584
5.4526
3.5328
2.5090
2.2918
2.2833
2.1254
1.0075
0.0075
0.0097
0.0942
0.7084
0.1801
[rel]
[ppm]

4e-^{13}CNMR

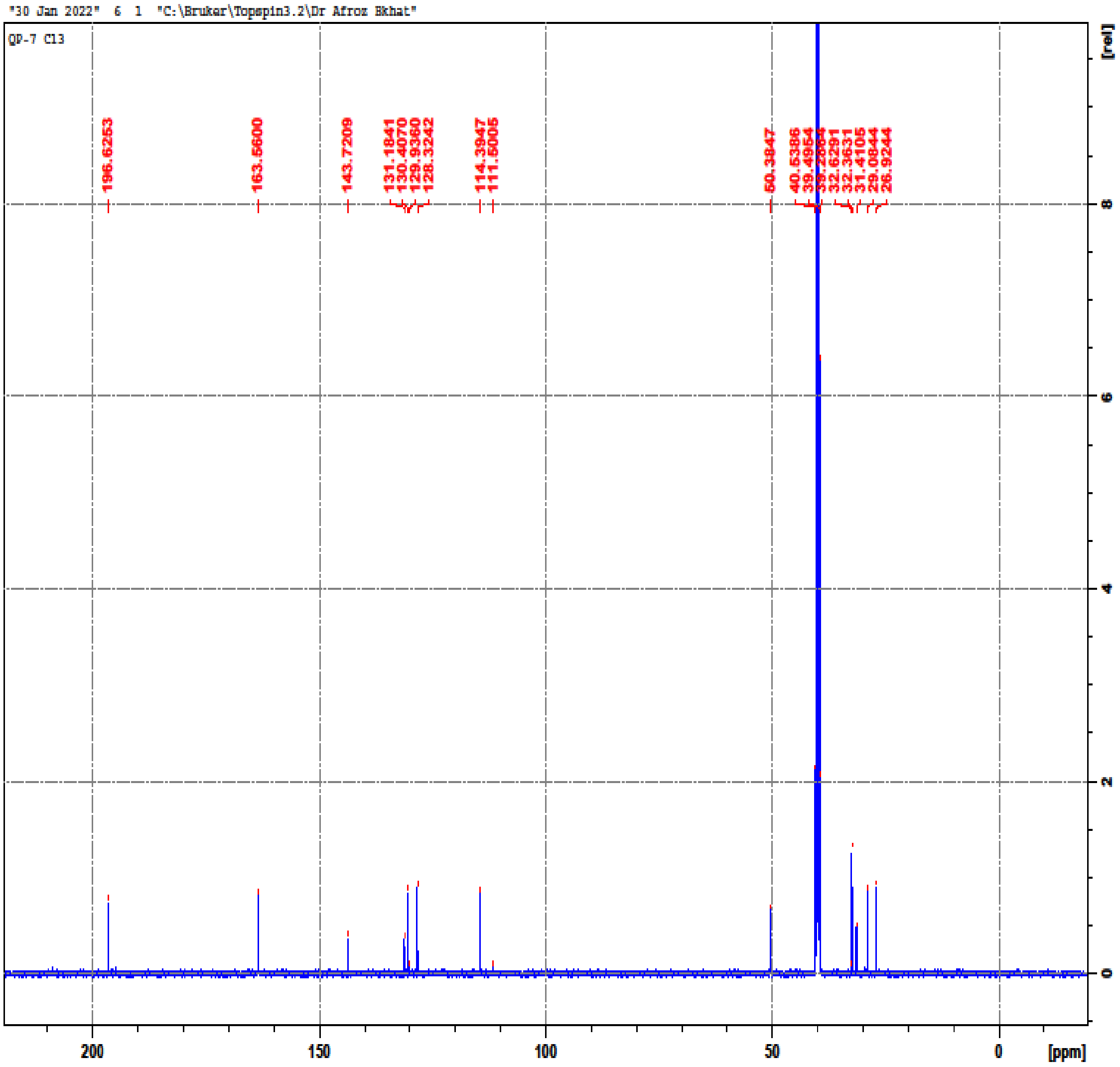
"30 Jan 2022" 6 1 "C:\Bruker\Topspin3.2\Dr Afroz Bkhat"
QP-7 C13
196.6253
163.5600
143.7209
131.1841
130.4070
129.9360
128.3242
114.3947
111.5005
50.3847
40.5386
39.4954
32.6291
32.3631
31.4105
29.0844
26.9244
[rel]
8
6
4
2
0
200
150
100
50
0
[ppm]

4e-HNMR

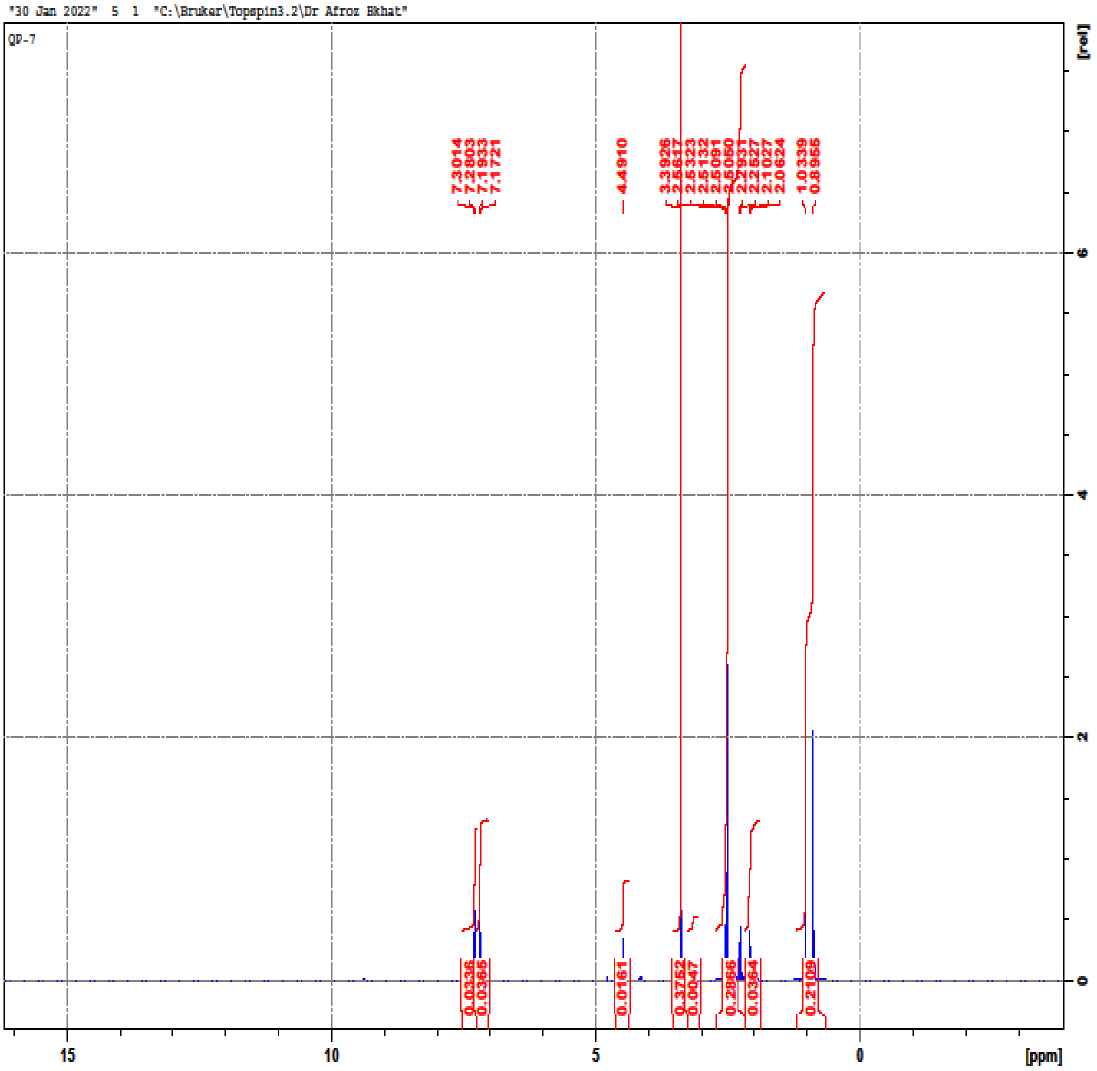
"30 Jan 2022" 5 1 "C:\Bruker\Topspin3.2\Dr Afroz Bkhat"
QP-7
7.3014
7.2803
7.1933
7.1721
4.4910
3.3926
2.5617
2.5323
2.5132
2.5091
2.5050
2.2931
2.2527
2.1027
2.0624
1.0339
0.8955
0.0336
0.0365
0.0161
0.3752
0.0047
0.2866
0.0364
0.2109
[rel]
6
4
2
0
15
10
5
0
[ppm]

4f-^{13}CNMR

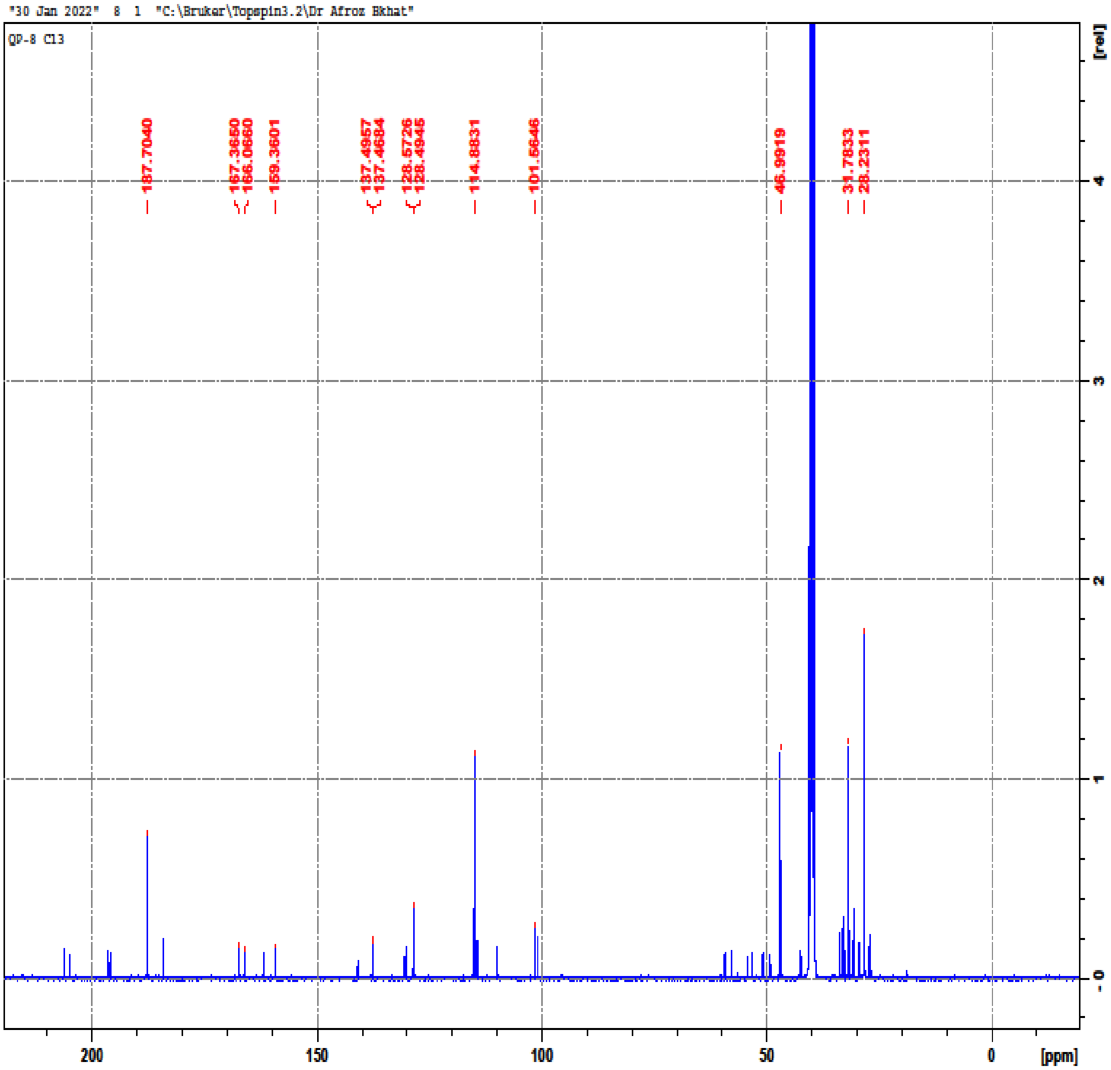
"30 Jan 2022" 8 1 "C:\Bruker\Topspin3.2\Dr Afroz Bkhat"
QP-8 C13
187.7040
167.3650
166.0660
159.3601
137.4957
137.4684
128.5726
128.4945
114.8831
101.5646
46.9919
31.7833
28.2311
[rel]
200
150
100
50
0
[ppm]

4f-HNMR

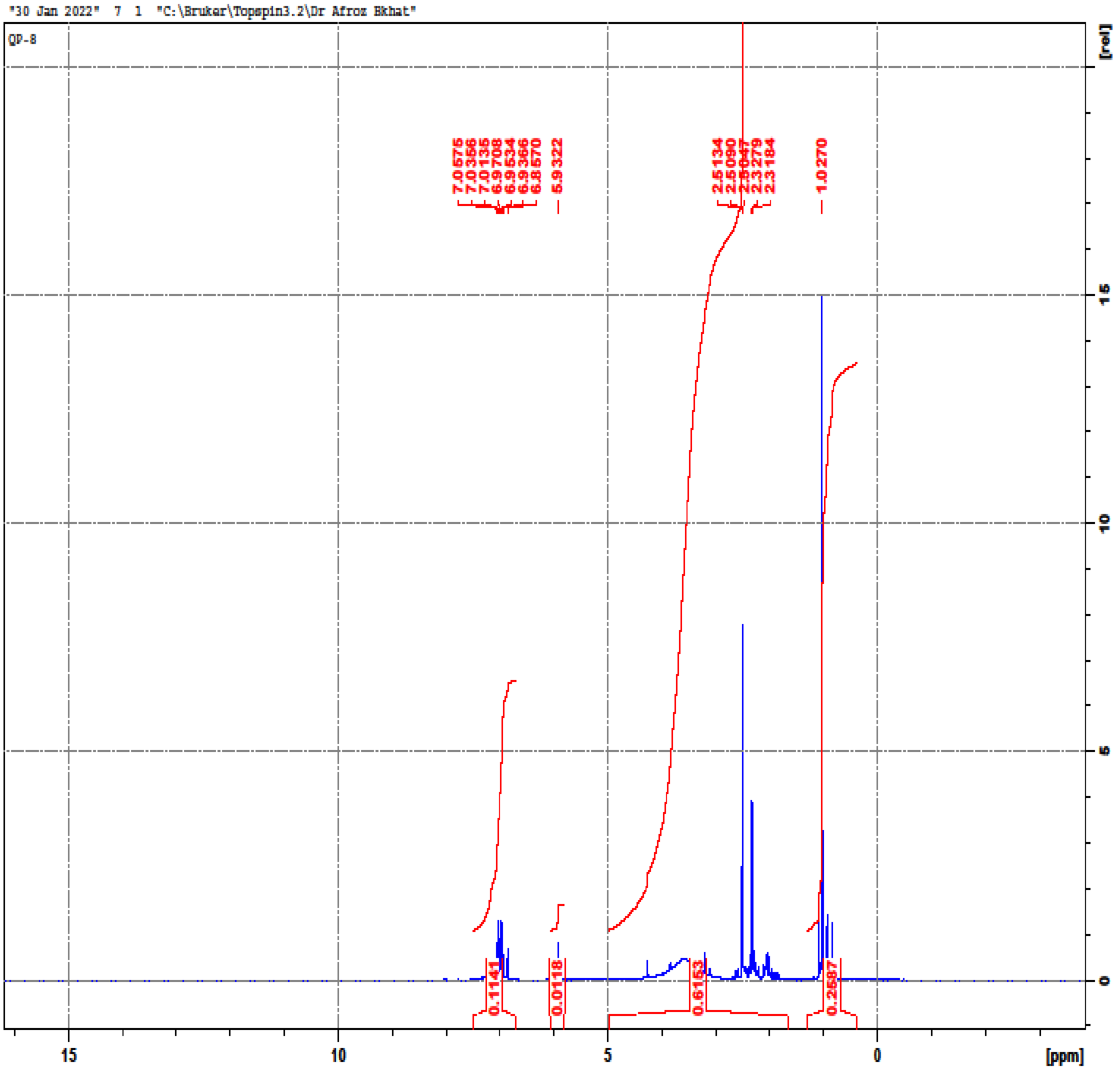

"30 Jan 2022" 7 1 "C:\Bruker\Topspin3.2\Dr Afroz Bkhat"
QP-8
7.0575
7.0356
7.0135
6.9708
6.9534
6.9366
6.8570
5.9322
2.5134
2.5090
2.5047
2.3279
2.3184
1.0270
0.1141
0.0118
0.6153
0.2587
[rel]
15
10
5
0
[ppm]

4g-^{13}CNMR

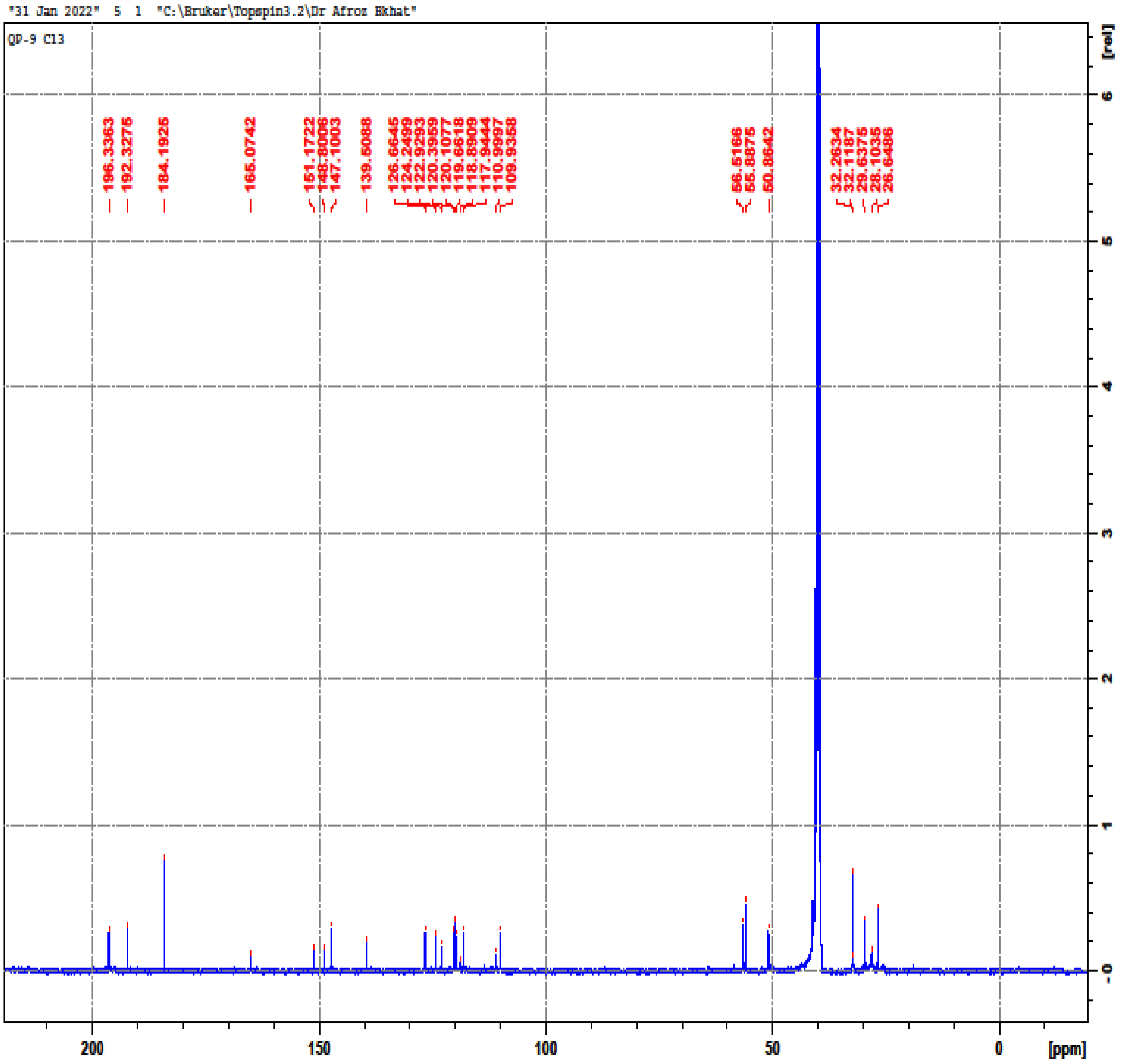
"31 Jan 2022" 5 1 "C:\Bruker\Topspin3.2\Dr Afroz Bkhat"
QP-9 C13
196.3363
192.3276
184.1925
165.0742
151.1722
148.8006
147.1003
139.5088
126.6645
124.2499
122.9293
120.3959
120.1077
119.6618
118.8909
117.9444
110.9997
109.9358
56.5166
55.8875
50.8642
32.2634
32.1187
29.6375
28.1035
26.6486
[rel]
6
5
4
3
2
1
0
200
150
100
50
0
[ppm]

4g-HNMR

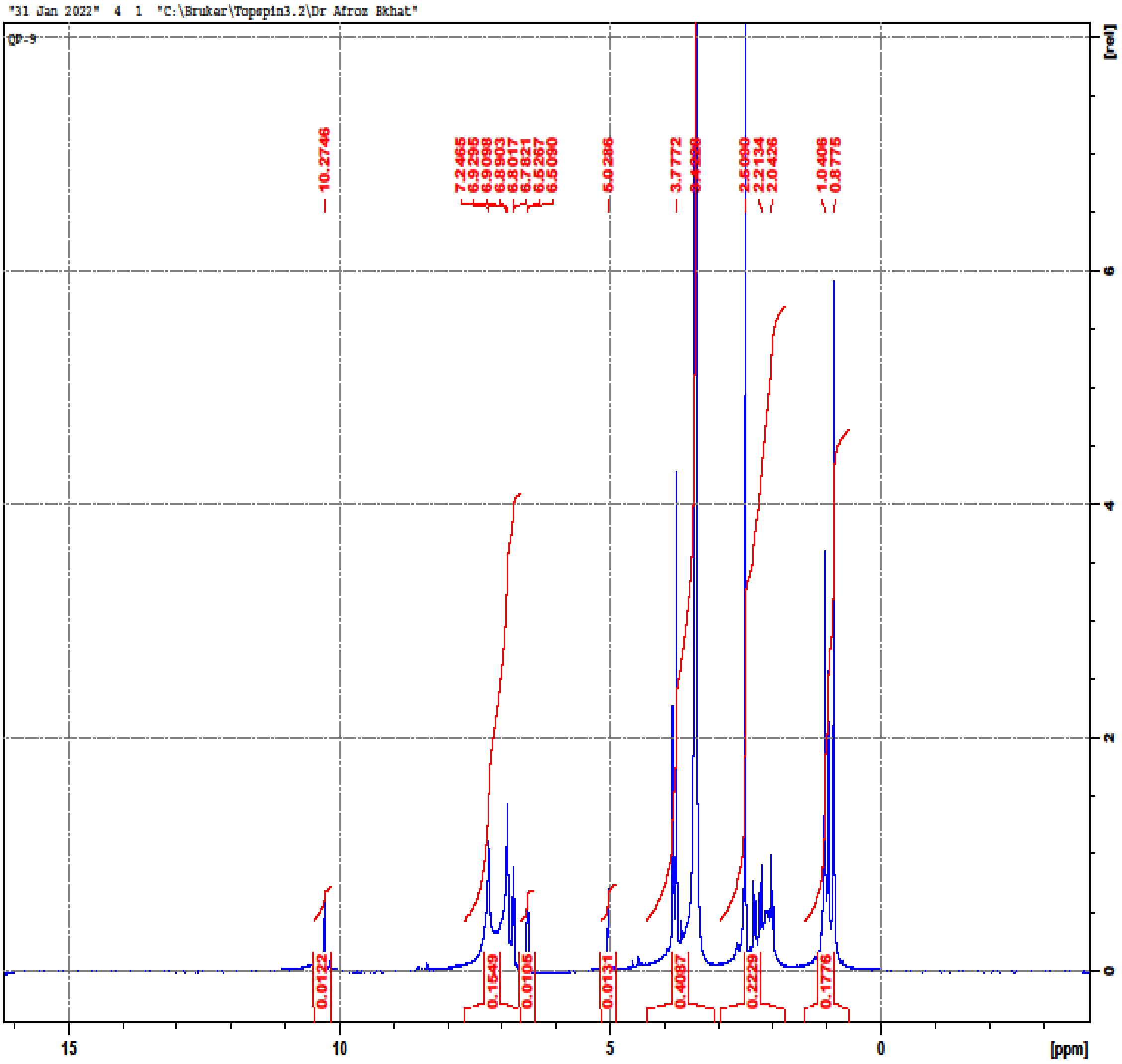
"31 Jan 2022" 4 1 "C:\Bruker\Topspin3.2\Dr Afroz Bkhat"
QP-9
10.2746
7.2465
6.9295
6.9098
6.8903
6.8017
6.7821
6.5267
6.5090
5.0286
3.7772
2.2134
2.0426
1.0406
0.8775
0.0122
0.1549
0.0106
0.0131
0.4087
0.2229
0.1776
15
10
5
0
[ppm]
[rel]
6
4
2
0

4h-^{13}CNMR

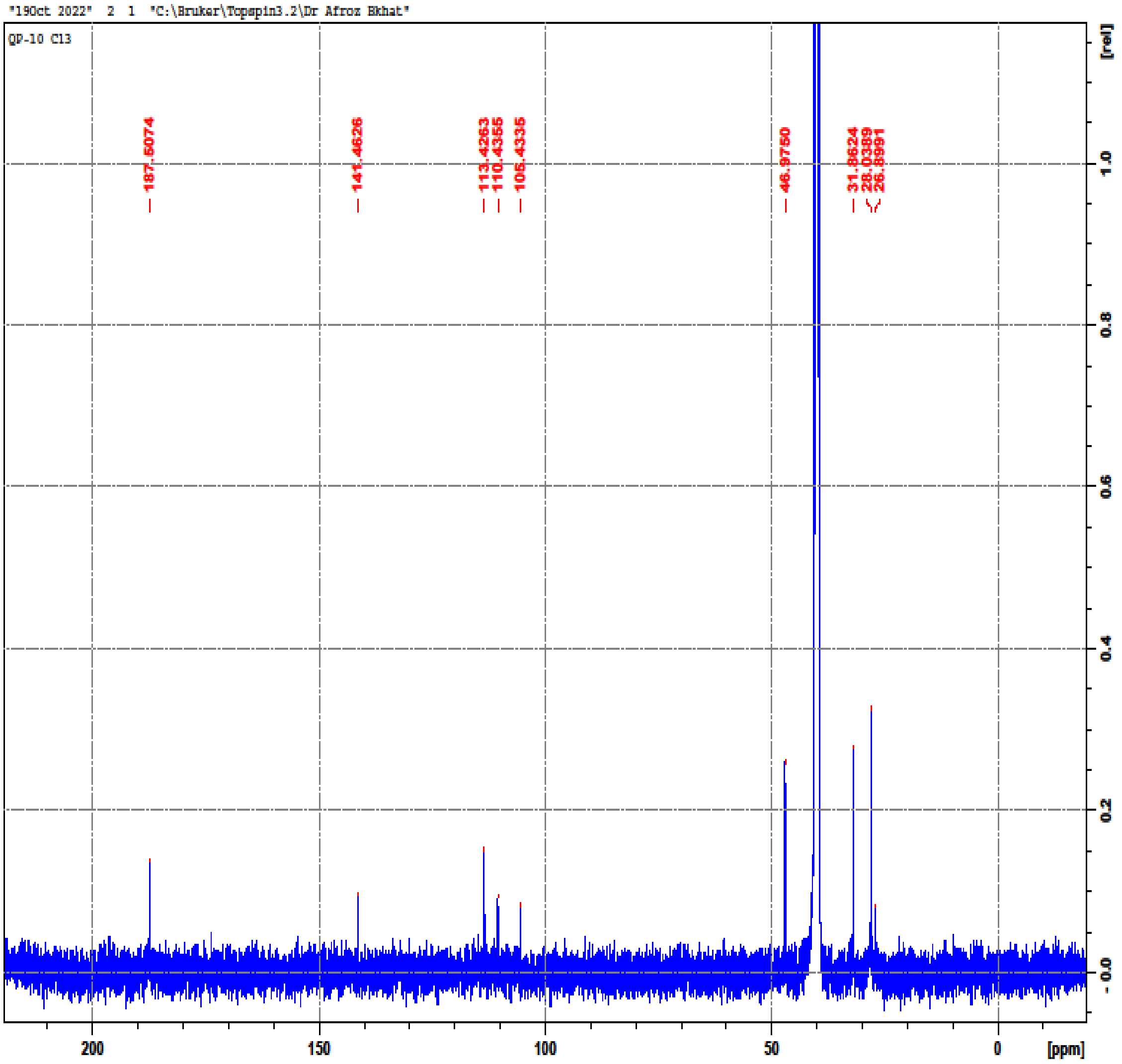

"19Oct 2022" 2 1 "C:\Bruker\Topspin3.2\Dr Afroz Bkhat"
QP-10 C13
187.5074
141.4626
113.4263
110.4355
105.4335
46.9750
31.8624
28.0389
26.8991
[rel]
1.0
0.8
0.6
0.4
0.2
0.0
200
150
100
50
0
[ppm]

4h-HNMR

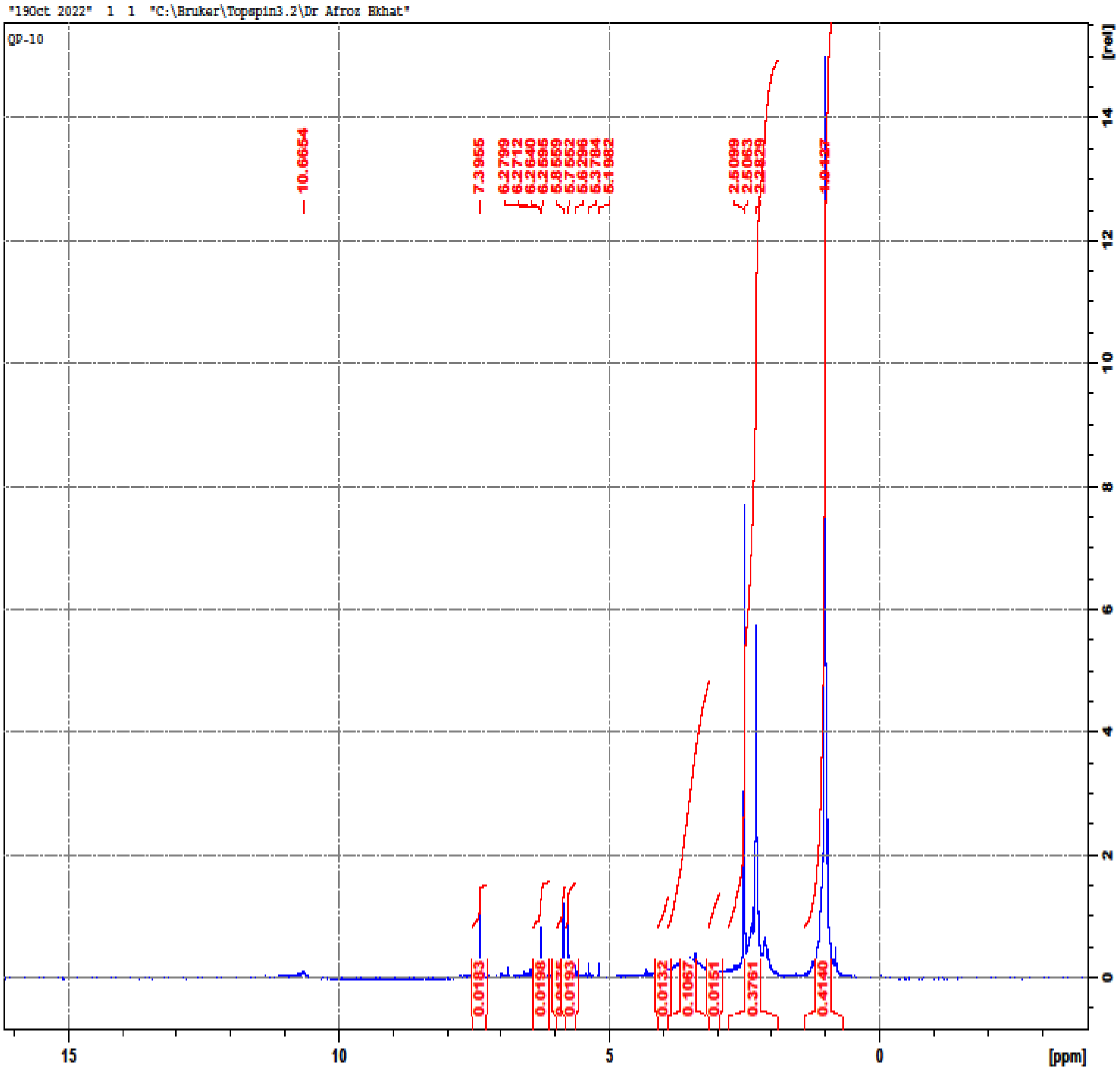
"19Oct 2022" 1 1 "C:\Bruker\Topspin3.2\Dr Afroz Bkhat"
QP-10
10.6654
7.3955
6.2799
6.2712
6.2640
6.2595
5.8559
5.7552
5.6296
5.3784
5.1982
2.5099
2.5063
0.0183
0.0198
0.0193
0.0132
0.1067
0.0151
0.3761
0.4140
[rel]
[ppm]
